# Auditory Cueing of Pre-Learned Skills and Role of Subcortical Information Processing to Maximize Rehabilitative Outcomes Bridging Science and Music-Based Interventions

**DOI:** 10.3390/healthcare10112207

**Published:** 2022-11-03

**Authors:** Concetta M. Tomaino

**Affiliations:** Institute for Music and Neurologic Function, Mount Vernon, NY 10552, USA; ctomaino@wartburg.org

**Keywords:** music therapy, neurologic music therapy, neurorehabilitation, subcortical networks, music and brain, music and health, cerebellum, music medicine

## Abstract

Auditory entrainment of motor function is a fundamental tool in neurologic music therapy with many studies demonstrating improved clinical outcomes in people with movement disorders such as Parkinson’s Disease, acquired brain injuries, and stroke. However, the specific mechanisms of action within neural networks and cortical regions that are aroused and influenced by auditory entrainment still need to be identified. This paper draws from some contemporary neuroscience studies that indicate the role of the cerebellum and other subcortical systems in modulating pre-learned motor schema and proposes a possible rationale for the success of auditory entrainment within neurologic music therapy.

## 1. Introduction Bridging Science and Music-Based Interventions

The field of music therapy encompasses a variety of clinical approaches in which music interventions are implemented to address specific treatment goals, e.g., psychological, developmental, rehabilitative, and medical. An area of music therapy that has seen the most growth and advancement in recent years has been neurologic music therapy (NMT) [1,2,3]. NMT utilizes evidence-based standardized music interventions based on data from translational neuroscience research to influence change in non-musical brain and behavioral function [3]. One of the most effective interventions for people with movement disorders, especially those with Parkinson’s disease (PD) is the use of rhythm to improve locomotion, balance, and gait [4,5,6,7]. The ability to track the actual beat (sound/silence) in rhythmic cueing in direct correlation to changes in gait (measured by changes in cadence, velocity, and stride length), has enabled research showing the efficacy of music-based treatments. NMT applies specific music-based cues, e.g., rhythm, and melody, to influence cognitive and motor responses in individuals with a range of brain-related injuries or chronic diseases. The rhythmic auditory cues often produce an immediate response that has been observed and documented in case studies and medical reports but often viewed as a fleeting phenomenon, e.g., the patients with movement disorders described in Oliver Sack’s book *Awakenings* who were immobile until music was played for them but returned to a rigid state when the music stopped [8]. However, prolonged engagement in precise music interventions has demonstrated improved function and carryover outside of music therapy [9,10,11].

Increased recognition of music therapy in neurologic rehabilitation has occurred in parallel with breakthroughs in neuroscience research of complex neural networks. Of special interest are studies of the influence of auditory signal processing in primary and secondary brain regions responsible for motor, speech, and cognitive functioning [2,12]. In fact, studies in music-making activities including singing and dancing reveal a strong coupling of perception and actions. Altenmüller and Schlaug (2013) suggest that this strong coupling is *mediated by sensory, motor, and multimodal brain regions and affects either in a top-down or bottom-up fashion important sound relay stations in the brain stem and thalamus* [1]. With the advances in neuroimaging and EEG technologies, it is becoming possible to study these complex systems, especially subcortical networks, in human subjects including those with brain injuries. The extensive neural network connections in the auditory system to motor networks from the spinal cord to the brain stem and upward to sensory–motor and supplementary motor cortices [9] allow auditory stimuli to quickly arouse and activate motor functions when internal systems may be impaired due to brain injury or neurological diseases. Although several papers have examined the principles of NMT in relation to neuroscientific studies [1,2,9,13,14], there is a need to study the underlying neural mechanisms and networks engaged by the auditory cueing used in NMT to effect positive clinical outcomes in those with impaired self-initiation and motor control.

## 2. Auditory Entrainment Is a Fundamental Agent of Action in NMT

Auditory motor entrainment is an essential component of neurologic music therapy. Entrainment is a process by which the signal or motion of one system synchronizes (entrains) to the motion of a second system. In NMT it is the auditory signal of rhythm, both the sound and pulse of the beat as well as the periodicity between the beats, that influence (entrain) an observable motor response synchronized to the auditory beat. In neurologic disorders, there are often fluctuations in the excitability of motor circuits thereby causing problems with physical motion and control. These fluctuations are greatly reduced when an external sensory cue provides the proper periodicity and modulation for the entrainment of internal motor network systems allowing for a continuous signal, which informs the flow, stability, and ease of movement [15].

Auditory entrainment can be the coupling of a specifically timed rhythmic beat to a motor response, e.g., walking and clapping, but can also be in the motor response to the duration of time between the beats. There is also evidence of the alignment of rhythmic sensory inputs to neural oscillations. A Neural Resonance Theory hypothesized by Large and Snyder [16] theorizes *that pulse and meter correspond to neural rhythms that synchronize with acoustic rhythms, influencing temporal expectancy, attention, and movement coordination.* Additionally, Large et al. [17] propose a model of beat perception neural networks for musical rhythms which provide a conceptual link between neural oscillations and induced pulse and meter in musical rhythm. Other studies indicate that these synchronized oscillations could act as a gating mechanism to quickly facilitate the proper routing of information between brain regions [18]. The regular external pulses presented to the brain have a driving effect on neural firing resulting in increased oscillatory coherence, which often occurs beyond cognitively tracked frequencies such as gamma (30–100 Hz)**.** This oscillatory coherence is imperative to neural connectivity, circuit function, and optimal function for a variety of health conditions [19].

### Engagement of Subcortical Systems by Auditory Entrainment Is Key Factor

The two subcortical systems known to be involved in timing and synchronization, are the cerebellum and the basal ganglia. Both are interconnected in processes related to motor timing and auditory cueing. Representation of timing information occurring in rhythmic synchronization and entrainment, such as pattern detection or tracking changes in rhythmic interval duration also takes place in the inferior colliculus (IC), which may serve a primary role in auditory–motor coupling during rhythmic entrainment [9]. The engagement of these subcortical regions in auditory cueing and motor coordination are important factors in the use of music-based interventions in neurorehabilitation.

The role of the cerebellum as a primary mechanism of action in informing motor timing function has long been established, and its strong connections to auditory networks are important to note. In his Theory on the Cerebral Cortex, from 1969, Marr [20] proposed that *the purpose of the cerebellum is to learn motor skills so that when they have been learned a simple or incomplete message from the cerebrum will suffice to provoke their execution*. Being able to stimulate pre-learned motor sequences through auditory coupling with cerebellar memory templates could explain the quickness at which auditory cueing can organize movement in people with neurologic disorders. By bypassing the need for thought-activated movement, motor sequencing and function occurs through habituated responses organized through the spinocerebellar tract, especially the vermis for externally paced motor responses [21]. For self-paced motor responses, the basal ganglia and supplementary motor areas are found to be more active [21]. In studying motor timing mechanisms in the cerebellum compared with the basal ganglia, Chauvigne et al. [21]. propose that the spinocerebellum plays a central role in auditory–motor entrainment while the cerebellum is more involved in mediating prediction and reducing error during motor tasks. A more recent review by Evers and Tölgyesi [22] explores the roles of different parts of the cerebellum for different aspects of music perception tasks along with individual connections to the basal ganglia. Other findings suggest that the entrainment circuit of the spinocerebellum can be stimulated by the auditory imagery of a pacing signal without, and external auditory stimuli if there is a previously associated auditory–motor schema for that action [23].

**Figure 1:**
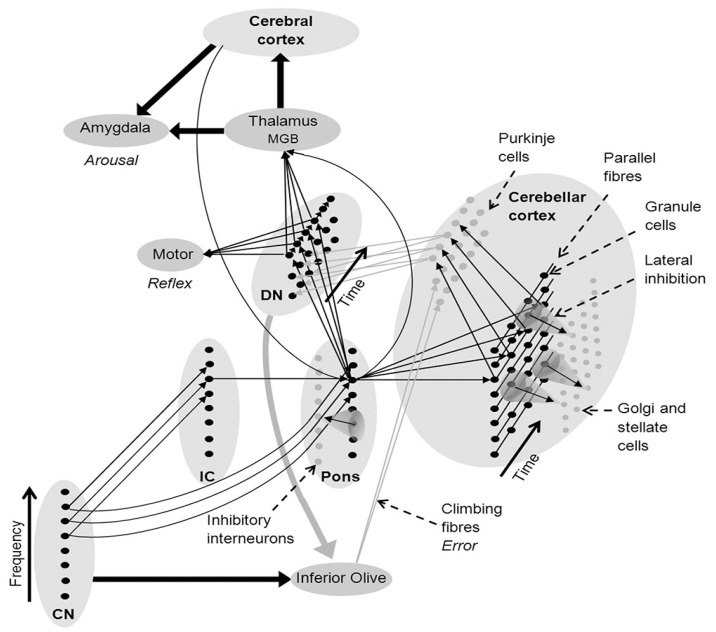
This image (from McLachlan, Wilson 2017) represents the connectivity network between cerebellum, brain stem, midbrain, and Cortex to support sound recognition and related auditory processes. Fine arrows denote neural connections involved in sound recognition and thick arrows denote connectivity between brain nuclei and regions (black denotes excitatory connections and gray inhibitory connections). CN, cochlear nucleus; DMGB, dorsal medial geniculate body; DN, deep nuclei of the cerebellum and IC, inferior colliculus.

As arousal of the brainstem and cerebellum by auditory stimuli occur early in auditory processing, informational patterns already formed as cerebellar templates allow for faster processing and integration into other functions such as motor timing and vocal communication. McLachlan and Wilson [24] point to the role of the pons, rather than the cerebral cortex, in the automatic processing of well-rehearsed sensory, cognitive, and motor function thereby reducing the role of the cerebellum in sound and speech processing to the recognition and learning of patterned sequences of pons inputs. This network of cortico–pons/cerebellar allows for multimodal templates to be associated with information then encoded in the cerebral cortex. McLachlan’s research shows that the cerebellum can play a major function in automatic motor responses in speech and movement from a bottom-up process—see Figure 1. He refers to this as *embodied cognition*, i.e., *in cerebellar circuits both the context and the rule are integrated in the same representation and thus undertake automatic processing of well-rehearsed sensory, cognitive, and motor function*. This enables the more efficient motor execution of pre-learned skills, especially when coupled with auditory-informed templates for movement and speech.

Yamazaki and Lennon [25] have proposed the cerebellum’s role in Reinforcement Learning (RL) and draws similarities to that of machine learning, i.e., making comparisons of the multiple areas of the cerebellum functioning as parallel processors. The speed at which the cerebellum can learn patterns for task execution, in absence of supervised learning further supports the important role it can have in allowing for preserved abilities (motor-related—including speech) to be activated when higher cortical processing has been damaged.

The role of the cerebellum in pre-learned motor tasks and its connections to auditory cueing is important because in some diseases, like Parkinson’s, beat perception may be impaired due to changes in the basal ganglia. Henry and Grahn [26] suggest that there is a neural synchrony between auditory and motor brain regions in the form of delta–beta phase–amplitude coupling might constitute a mechanism for functional auditory–motor coupling. Research in beat perception indicates that neural oscillations with frequencies corresponding to the stimulus (beat) rate are most likely in the delta frequency band (0.5–4 Hz or 30–240 beats per minute), which encompasses the range of rates within which the sense of the beat is felt [26]. Fries [27] points to the effect of synchronization on communication between neuronal groups, specifically that Gamma-band (30–90 Hz) synchronization occurs rapidly enough to activate postsynaptic neurons effectively to escape the following inhibition. He refers to this as *communication through coherence*. The optimization of neural oscillation coherence as a result of auditory entrainment can enhance motor function outcomes when top-down processes are slowed or damaged by acquired or chronic brain injuries.

Brown et al. [28] evaluated auditory timing in motor coordination in relation to dance. During the auditory-motor entrainment, in this context, auditory input is relayed to key subcortical structures via the medial geniculate nucleus of the thalamus. The cerebellum, particularly the anterior vermis and lobules V and VI, together with the thalamus, form a circuit that plays an important role in the automaticity of musically entrained movements. In parallel, the thalamus also interacts with the basal ganglia (especially the putamen), which together with connections to the motor cortices in corticobasal ganglia-thalamocortical loops, functions in the selection and sequencing of particular action segments, especially in the context of the temporally regular/predictable movement cycles that characterize movement to music.

In sum, there is strong evidence that musical stimulation can act via subcortical structures to provide access to a widespread network of cortical and subcortical processing involved in limb movement, potentially aiding rehabilitation in the face of damage localized to specific portions of this network’s cortical extent.

## 3. Discussion: Implications for Neurologic Music Therapy in Neurorehabilitation

Music processing arouses and recruits multiple levels of neural interconnectivity from the most basic arousal mechanisms to complex higher cortical processes [29,30] enabling it to stimulate and engage residual or pre-learned habituated function. This is especially important to consider in designing music-based therapies to aid those who have difficulty in motor planning tasks and initiation of motor activity. Research in music perception has shown that the structure of rhythm (patterned generated sound) provides a framework that informs time-ordered activities as well as enhances memory retention and recall [31,32]. Auditory rhythmic cueing is an efficacious tool because it can engage areas of the brain, like the cerebellum, that do not rely on high-order mental processing to execute pre-learned skills.

McLachlan and Wilson’s concept of embodied cognition can also help explain why auditory entrainment can arouse and stimulate pre-learned patterns of motor responses in persons with movement disorders. Individuals with neurologic impairments such as stroke or traumatic brain injuries may have challenges in executive function, including initiation and multi-step planning, making the coordination of movement difficult from a top-down perspective. However, by arousing pre-learned patterns of motor response—arousal of the cerebellar circuits via auditory pathways can bypass the need for the patient to think through how to move, especially when brain injury makes it difficult to do so independently.

For people with neuromuscular motor impairments, it is the self-pacing and self-directed motor timing that is impaired. For this reason, the importance of the spinocerebellum auditory coupling with motor networks needs to be considered, as it enables the preserved motor function to take place from bottom-up processes. In relation to the use of rhythmic entrainment within rehabilitation, research indicates that it is period (interval-based) entrainment rather than beat entrainment that is most effective in providing a template for motor planning and motor execution response [5]. This distinction becomes important when the type of auditory cue is chosen to affect a response in a person with motor issues related to gait, coordination, or speech production. It is also important to note that many patients undergoing neurorehabilitation have challenges with cognitive processes, especially executive function, and motor planning. In most instances, continuous attention is necessary for new learning or relearning of skills to take place, but for those with progressive diseases such as Parkinson’s and Multiple Sclerosis, or in acquired brain injuries, it is the engagement time of the motor activity that will carry over even when there is cognitive impairment. The evidence is that the temporal patterns of sound in conjunction with embodied cognition of movement patterns in the cerebellum allow for the successful execution of motor functions.

Although many clinical studies identify successful music-based intervention for people with neuromotor impairments [33,34,35,36] understanding the important role of auditory stimulation of subcortical networks to activate learned automatic responses is still needed. Neuroscience studies by Särkämo and colleagues indicate that music listening activates broad bilateral networks involved in memory, motor function, emotional regulation, and attention with significant enhancement in the domains of verbal memory and focused attention [37]. Similarly, consistent exposure to autobiographical music can improve cognitive function in those with neurocognitive deficits by activating and improving distinct neural networks that process both aspects of music and memory, particularly the prefrontal cortex [38].

EEG and Neuroimaging studies of finger tapping have provided preliminary evidence of expectation, priming, and entrainment in frontal, temporal, and supplemental motor cortices. In PD, as mentioned previously, the changes in basal-ganglia-cortical networks are associated with alterations in timing perception and the production of rhythmic-based events—especially movement. Given the quickness at which the external auditory cue can excite cerebellum and premotor areas into action—the schema for motor function is initiated, thus enhancing gait and reducing freezing or shuffling in those with conditions like Parkinson’s Disease. Improved, too, are balance, stride length, posture, and side–side movement, as well as complex coordinated movement sequences combining upper and lower limbs [39,40].

The use of auditory rhythmic stimuli to inform or provide templates for habituated motor function including motor speech function is equally effective for those with acquired brain injuries, including stroke [38], and often with better outcomes and carryover than traditional motor rehabilitation due to the focus on subcortically driven activity driven by auditory stimulation than more conscience and intent motor planning, which requires higher cognitive processing [1,40,41].

## 4. Conclusions

The research showing that the cerebellum and subcortical networks are capable of mediating pre-learned/habituated motor activity and that these brain regions can be entrained by auditory stimulation provides a rationale for the efficacy of many techniques within neurologic music therapy. In Sensorimotor Rehabilitation the music/rhythm-based intervention techniques most often used are Rhythmic Auditory Stimulation (RAS), Patterned Sensory Enhancement (PSE), and Therapeutic Instrumental Music Performance (TIMP). In speech and language rehabilitation, the following auditory-based techniques are used: Melodic Intonation Therapy (MIT); Musical Speech Stimulation (MUSTIM); Rhythmic Speech Cueing (RSC); and Oral Motor and Respiratory Exercises (OMREX). All the above techniques rely on the pattern of rhythm and contour of sound to inform pre-learned skills in those with a neurologic rehabilitation goal. Evidence of the role of auditory stimulation on arousal and entrainment of subcortical systems especially the cerebellum provides a rationale for the efficacy of neurologic music therapy techniques. The pattern information provided by auditory cues can inform motor, attention, and learning even when higher cognitive and motor planning areas have been damaged. This is the gateway point to the recovery of function and the principal reason why auditory entrainment is an effective rehabilitation tool in neurologic music therapy.

The music therapist needs to understand the neuroscience of the human brain and how diseases and trauma impact neurologic function. Needed, too, is knowledge of music perception and of how music stimuli impact arousal and the organization of various brain systems. Continued research on the mechanisms of action in auditory entrainment of subcortical systems in neurorehabilitation will enhance the incorporation of music-based interventions into standard rehabilitation practice.

## Data Availability

Not applicable.
